# Necessity of Attention to Mental Health of the Front Line Nurses against COVID-19: A Forgotten Requirement

**DOI:** 10.30476/IJCBNM.2020.85889.1301

**Published:** 2020-07

**Authors:** Majid Purabdollah, Mostafa Ghasempour

**Affiliations:** Department of Nursing Education, School of Nursing and Midwifery, Tabriz University of Medical Sciences, Tabriz, Iran

**DEAR EDITOR**

Today, emergence of infectious diseases has overshadowed many health beliefs and played a great role in the history of health development. The United Nations Secretary-General has introduced “Communicable diseases” as one of the challenges of our time. Viruses can be one of the causes of contagious diseases. The Coronavirus disease 2019 (COVID-19) outbreak, recently launched from Wuhan, China, has spread to more than two-thirds of the world, including Iran, and has reached the pandemic stage. ^1^ In particular, COVID-19 disease further affects the healthcare sector and leads to many challenges for this important part of society.These challenges include increasing need for medical staff; increasing costs for preventive and personal protective equipment for personnel, diagnostic and laboratory costs and treatment costs; increasing the need for Intensive Care Unit (ICU) beds and ventilators; and increasing mortality. ^1^

In the meantime, health care professionals play an important role in managing such crises. Nurses, as the largest group of health care workers, spend more time with patients than other healthcare professionals and play an important role in the care, control and treatment of these diseases. During the epidemic of emerging diseases, all social organizations, even the patients’ family members, are distanced from him/her, and it is the duty of the medical staff to take care of the patient despite the potential health risks. ^2^ To understand the significance of the issue, it is sufficient to know that about 50% of those who died in the Severe Acute Respiratory Syndrome (SARS) coronavirus epidemic were health workers who were somehow exposed to patients in the hospital. ^3^

Past studies show that mental disorders, including post-traumatic stress disorders, anxiety, depression, panic attacks, irritability, delirium, mistrust, and even suicidal thoughts, were prevalent among nurses who cared for patients with SARS. Developed mental disorders can lead to psychological disorders, loss of appetite, fatigue, impaired physical ability, sleep disorders, irritability, apathy, numbness, fear, and hopelessness. ^4^

In accordance with the ethical Reciprocity Principle, hospitals have a reciprocal duty towards the health care staff. Health facilities should provide the necessary infra-structure to support the staff in the care of patients with emerging diseases, including communicating with the staff to control infection, providing sufficient support to increase the staff motivation, providing personal protective equipment, and especially psychological screening for nurses, psychiatric counseling and psychological support. ^2^ According to a recent report by the National Federation of Nurses in Italy, the second Italian nurse has committed suicide after being informed of a positive corona virus test. ^5^ Therefore, the importance of paying attention to the psychological issues of nurses during and after caring for patients with COVID-19 is obvious.

However, due to the increasing prevalence of the virus and also the increasing number of deaths of nurses, which can be a stressor for other nurses, it is necessary to pay attention to mental health of nurses. Stress and mental disorders can, like a vicious cycle, weaken the immune system and lead to coronavirus infection, especially where nurses with a history of mental disorders are more concerned. Nurses’ mental disorders can also significantly reduce their quality of care. Despite the major importance of this issue and the findings of previous studies, this issue has not been addressed yet. Therefore, it is imperative to pay attention to the mental and psychological issues of nurses in the care of patients with coronavirus 19 as the current priority of care for nurses. In this regard, experiences and measures can be used in the epidemic of SARS, Middle East Respiratory Syndrome-related coronavirus (MERS) and Ebola patients, including screening of nurses for mental health, psychological counseling, or training courses to provide comprehensive and patient-centered care. 
